# Updated foxtail millet genome assembly and gene mapping of nine key agronomic traits by resequencing a RIL population

**DOI:** 10.1093/gigascience/giw005

**Published:** 2017-01-20

**Authors:** Xuemei Ni, Qiuju Xia, Houbao Zhang, Shu Cheng, Hui Li, Guangyu Fan, Tao Guo, Ping Huang, Haitao Xiang, Qingchun Chen, Ning Li, Hongfeng Zou, Xuemei Cai, Xuejing Lei, Xiaoming Wang, Chengshu Zhou, Zhihai Zhao, Gengyun Zhang, Guohua Du, Wei Cai, Zhiwu Quan

**Affiliations:** 1 BGI-Shenzhen, Shenzhen 518083, China; 2 Institute of Millet, Zhangjiakou Academy of Agricultural Science, Zhangjiakou 075000, China; 3 State Key Laboratory of Agricultural Genomics, BGI-Shenzhen, Shenzhen 518083, China; 4 Key Lab of Genomics,Chinese Ministry of Agriculture, BGI-Shenzhen, Shenzhen 518083, China; 5 Guangdong Province Key Laboratory of Crop Germplasm Research and Application, BGI-Shenzhen, Shenzhen 518083, China; 6 Shenzhen Engineering Laboratory of Molecular Design Breeding, BGI-Shenzhen, Shenzhen 518083, China; 7 BGI Millet Co., Ltd, BGI-Shenzhen, Shenzhen, 518083, China

## Abstract

Foxtail millet (*Setaria italica*) provides food and fodder in semi-arid regions and infertile land. Resequencing of 184 foxtail millet recombinant inbred lines (RILs) was carried out to aid essential research on foxtail millet improvement. A total 483 414 single nucleotide polymorphisms were determined. Bin maps were constructed based on the RILs’ recombination data. Based on the high-density bin map, we updated Zhanggu reference with 416 Mb after adding 16 Mb unanchored scaffolds and Yugu reference with some assembly error correction and 3158 gaps filled. Quantitative trait loci (QTL) mapping of nine agronomic traits was done based on this RIL population, five of which were controlled by a single gene. Meanwhile, two QTLs were found for plant height, and a candidate gene showed 89% identity to the known rice gibberellin-synthesis gene *sd1.* Three QTLs were found for the trait of heading date. The whole genome resequencing and QTL mapping provided important tools for foxtail millet research and breeding. Resequencing of the RILs could also provide an effective way for high-quality genome assembly and gene identification.

## Introduction

Foxtail millet (*Setaria italica*) was an ancient cultivated crop domesticated in China more than 8700 years ago [1, 2]. It provided the most important food and forage for the Yellow River valley in ancient China and is still an essential food source in semi-arid areas [3, 4]. Although foxtail millet is one of the most drought-tolerant crops, the low productivity of unimproved foxtail millet has limited its application in agriculture. In the past decade, high-yield hybrids with herbicide-resistance that can dramatically improve crop production and decrease labor cost were inbred, indicating that foxtail millet had the potential for becoming a high-yield crop through the use of genetic tools [5–7].

Foxtail millet genome *de novo* sequencing was initially finished in 2012 [8, 9]; here we carried out resequencing of foxtail millet recombinant inbred line (RIL) population and construction of the high-resolution bin map. A high accuracy Zhanggu millet genome reference (anchoring 96% scaffold sequence) was constructed using the high-resolution bin map based on the previous Zhanggu draft genome [9]. Additionally, this bin map was also used to check the assembly error in the Yugu genome reference [8], and a second edition (anchoring 99.7% scaffold sequence) was done after assembly error correction and gap filling. Nine agronomic traits were phenotyped in 2010 and 2011, respectively. Quantitative trait loci (QTL) analysis was done based on genotype and phenotype data, and some loci were mapped at a 130-kb region using these 184 RILs, indicating that resequencing of foxtail millet RIL population could provide an effective approach for high-quality genome assembly and gene mapping. The genome reference and single nucleotide polymorphism (SNP) markers will become an important tool in foxtail millet molecular breeding, and the loci related to the nine agronomic traits will provide pivotal information to breeders.

## Materials and methods

No specific permissions were required for the described field studies. The location is not privately owned or protected, and the field studies did not involve endangered or protected species.

### Sampling and RIL construction

Zhanggu was selected as the male parent line and A2, a popular male sterile line that has been used widely for hybrid breeding, was selected as the female parent line. F1 was constructed from a cross between Zhanggu and A2, and RILs were developed using single seed descent strategy. The segregation population was grown three generations per year in New Village, Jiyang Town, Sanya City, Hainan province (coordinates: 109°35΄E/18°17΄N) (November to January; January to April) and Erliban Village, Shalingzi Town, Xuanhua County, Zhangjiakou City, Hebei province (coordinates: 114°54΄E/40°40΄N) (May to October).

### Phenotyping

Nine agronomic traits contained flag leaf length, leaf color, bristle color, anther color, plant height, ear height, heading date, panicle hardness, and sehtoxydim resistant.

The male parent Zhanggu had green leaf, red bristle, yellow anther with sethoxydim-resistant, while the A2 had yellow leaf, green bristle, brown anther with sethoxydim-sensitive. F1 had green leaf, red bristle, brown anther with sethoxydim-resistant.

All the color traits were characterized according to the RILs’ appearance of different development periods. Leaf color was observed and recorded as yellow or green 15 days after sowing. The red or green bristle color was collected 10 days after heading, and anther color was recorded as yellow or brown during flowing period.

Heading date was recorded as the number of days from sowing to heading. Flag leaf length and width were measured at the maximal values for each flag leaf using a ruler. Plant height was measured the distance between panicle terminal and ground. Additionally, ear height was measured as the distance between flag leaf ear and ground. Panicle hardness was observed and recorded as stiff or flexible 30 days after flowering.

Sethoxydim-resistant data were collected after 3 days seeding on sethoxydim medium (50 mg/L), normal growing recording resistant, and wilting or dead recording sensitive.

### DNA isolation and genome sequencing

Total genomic DNA was extracted from young leaf tissues of F10 using the CTAB method [10]. DNA was quantified using 1% agarose gel electrophoresis and Qubit Fluorometer. Then 500-bp pair-end libraries were constructed under the standard protocol provided by Illumina (San Diego, CA, USA). The sequencing was performed using Hiseq2000 for pair-end 50-cycle sequencing according to the manufacturer's standard protocol. Low-quality reads, reads with adaptor sequences, and duplicated reads were filtered and the remaining high-quality data were used in SNP calling.

### Sequence alignment, genotyping, and recombination breakpoint determination

Reads of all samples were mapped onto Zhanggu initial genome reference (containing nine pseudo chromosomes and unmapped scaffolds) by using SOAP2 [11] (vision 2.20). The input data for SNP calling was prepared by SAMtools [11] (version 0.1.8) and then SNP calling was conducted by realSFS (version 0.983), based on the Bayesian estimation of site frequency at each site. The SNPs for further analysis were selected by the following criteria: the different alleles between two parents with missing data <60%. Then a sliding window approach was used to evaluate 15 consecutive SNPs for genotype calling and continued the process as the window slide base-by-base [12]. This approach was adopted in several studies, and we followed their methods for genotyping identification [12, 13]. The window with a Zhanggu:A2 SNP ratio of 11:4 or higher was called Zhanggu genotype, 4:11 or lower called A2 genotype, and SNP ratio between 11:4 and 4:11 was called heterozygous. The breakpoint was determined at the boundary of the Zhanggu, A2, and heterozygous.

### Bin map and chromosome construction

All breakpoints were aligned along the Zhanggu initial chromosomes sorted from the upper end to bottom end with 20-kb minimal intervals. Adjacent intervals with the same genotype across the 184 RILs were defined as a single recombination bin [14]. The recombination bins were serving as genetic markers, and the linkage map was constructed using MSTMap with Kosambi's mapping function (http://alumni.cs.ucr.edu/∼yonghui/mstmap.html). Then the Zhanggu genome was updated based on bin map.

### Gene mapping and QTL mapping

The phenotype of each RIL and genotype of each bin were collected for gene mapping and QTL analysis. QTLs were identified using composite interval mapping performed in the software package MapQTL 5 [15].The likelihood ratio statistic was computed for every bin, and QTL were called for LOD values >3.0.

### Yugu genome construction and gap filling

Reads of A2 and RILs were mapping to the chromosomes and scaffolds of Yugu [8]. The SNP calling procedure was the same as the above method. The differences were that we could not identify the Zhanggu allele directly. So we imputated the male parent allele according the population SNPs, that is, SNP identical to A2 was considered as A2 allele and contrary to A2 was considered as Zhanggu allele. The bin map was constructed using the same strategy as before, where bins with abnormal linkage were moved to the proper position according to the bin map. Gaps with two flank sequences were mapped to the Zhanggu genome. A gap was filled with the Zhanggu sequence only when both flank sequences were matched to the Zhanggu sequence. The Zhanggu sequence filled in the Yugu gap was shown in lowercase (Fig. 2).

## Results

**Figure 1.**
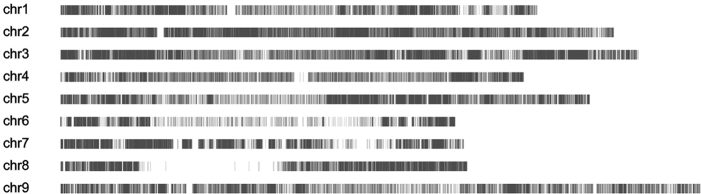
Distribution of 483 414 SNPs between Zhanggu and A2.

A total of 140 Gb clean data was generated, which gave a 2× depth of each RIL on average. A total of 483 414 SNPs were detected, given an average density of 1.2 SNPs/kb for the RILs (Table 1, Fig. 1).

**Table 1.** Summary of the bins and SNP distribution in Zhanggu

Chromosome	Length (bp)	Bin number	Linkage (cM)	SNP number	SNP density (/kb)
chr1	44 603 498	408	167.117	42 624	0.955
chr2	51 761 675	491	224.647	89 674	1.732
chr3	54 090 027	396	221.738	81 236	1.502
chr4	43 349 090	348	168.232	27 932	0.644
chr5	49 560 508	480	267.52	60 305	1.217
chr6	36 928 436	203	152.1	30 946	0.838
chr7	37 743 793	242	168.48	56 045	1.485
chr8	38 066 565	283	205.431	58 149	1.528
chr9	59 875 680	586	352.58	36 503	0.609
Total	415 979 272	3437	1927.845	483 414	1.162

**Figure 2.**
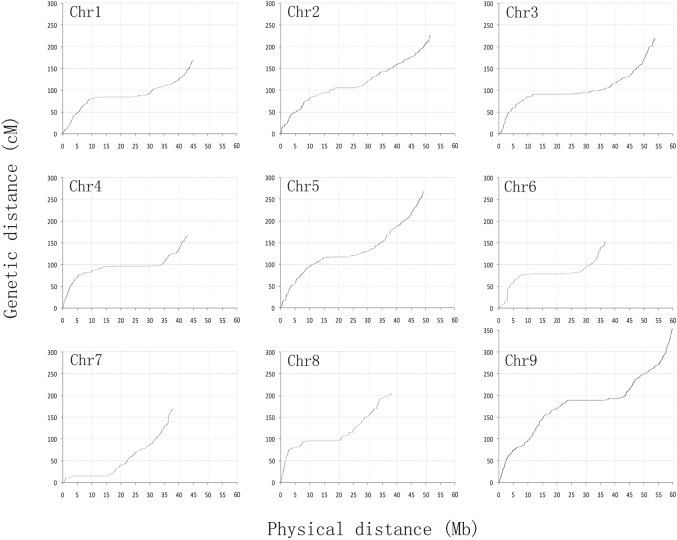
Genetic distance vs. physical distance. Genetic position of the 3437 bins is plotted against the corresponding physical position. Regions with low ratio of genetic distance to physical distance show heterochromatin regions.

**Figure 3.**
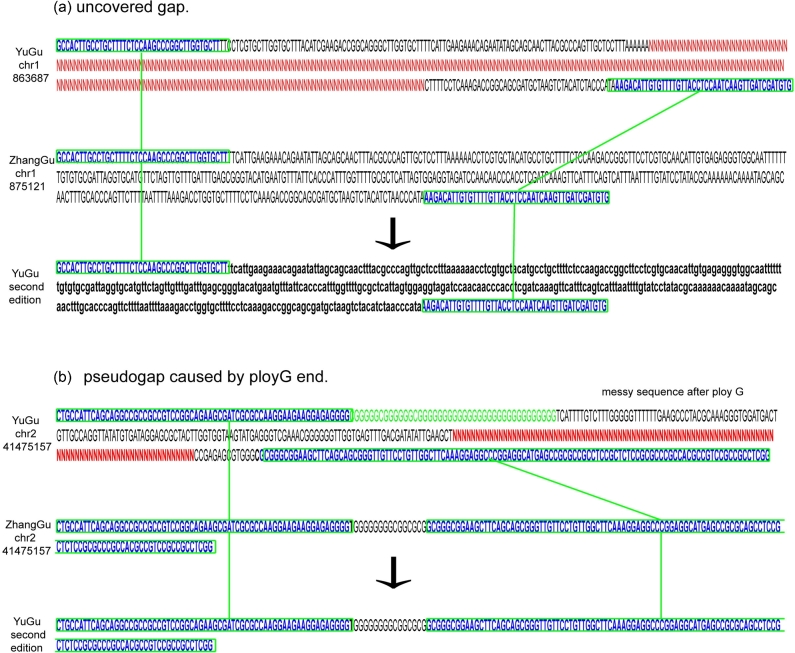
Gap filling in Yugu chromosome. Gap was filled by the Zhanggu sequence when both flank sequences were matched to the Zhanggu chromosome. (**a**) Gap was caused by low coverage, both flank sequences were matched to the Zhanggu, gap was filled by the lowercase Zhanggu sequence. (**b**) Gap was caused by ploy G sequence, Sanger sequencing cannot step over the ploy G.

**Figure 4.**
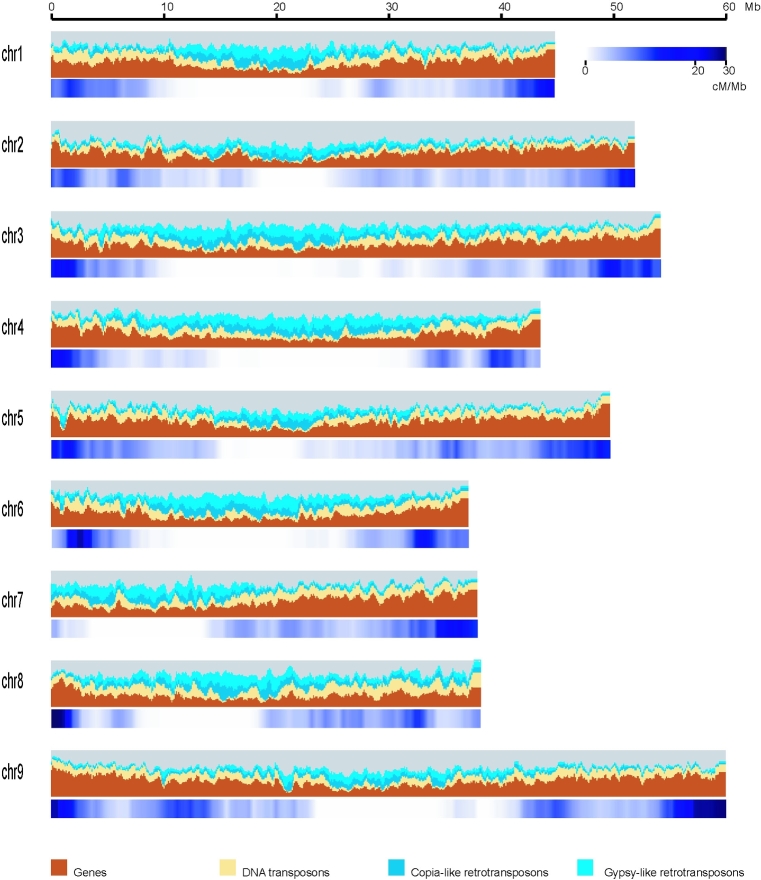
Genomic landscape of the Zhanggu chromosomes (second edition). Major DNA components are categorized into genes (brown), DNA transposons (yellow), Copia-like retrotransposons (dark blue), Gypsy-like retrotransposons (light blue), with respective DNA contents of 19%, 13%, 10%, and 21% of the genome sequence. Categories were determined for 1-Mb windows with a 0.2-Mb shift. Recombination ratio was shown in blue bars, ranging from 0 cM/Mb to 30 cM/Mb.

We identified 3437 recombination bins in the 184 RILs, the physical length of the recombination bins ranging from 20 kb to 12 Mb, given an average length of 121 kb (Table S1). Nine linkage groups, with a total genetic distance of 1927.8 cM (Table 1, Fig. 2), were constructed. The interval between these bins ranged from 0.1 cM to 13.8 cM, with an average of 0.56 cM. Chromosomes were updated based on the bin map and scaffolds of Zhanggu, and the second edition Zhanggu reference genome was generated (416 Mb) after adding unanchored scaffolds (16 Mb) (Table 2, Fig. 4). The assembly errors of Yugu genome reference were revised based on the bin map constructed by alignment on Yugu genome. A total of 3158 gaps was filled by Zhanggu sequences using sequence homology BLAST. The second edition Yugu reference genome was constructed after assembly error correction and gap filling (Fig. 3).

**Table 2.** Summary of Zhanggu second edition and Yugu second edition

Strain	Chromosome length (bp)	Gap length (bp)	Gap number	Gap ratio	Filled gap number
Zhanggu	399 854 594	26 817 695	31 942	6.7%	/
Zhanggu^2th^	415 979 272	28 962 873	34 452	7.0%	/
Yugu	401 300 876	4 616 102	6171	1.2%	/
Yugu^2th^	402 520 233	2 175 332	3297	0.5%	2874

To identify agronomic traits related loci that were important in foxtail millet, QTL analysis was done based on this RIL population [14, 16]. Nine agronomic traits, which can be divided into two categories: qualitative traits (sethoxydim resistance, leaf color, bristle color, anther color, panicle hardness) and quantitative traits (plant height, heading date, flag leaf width, flag leaf length) [17].

**Figure 5.**
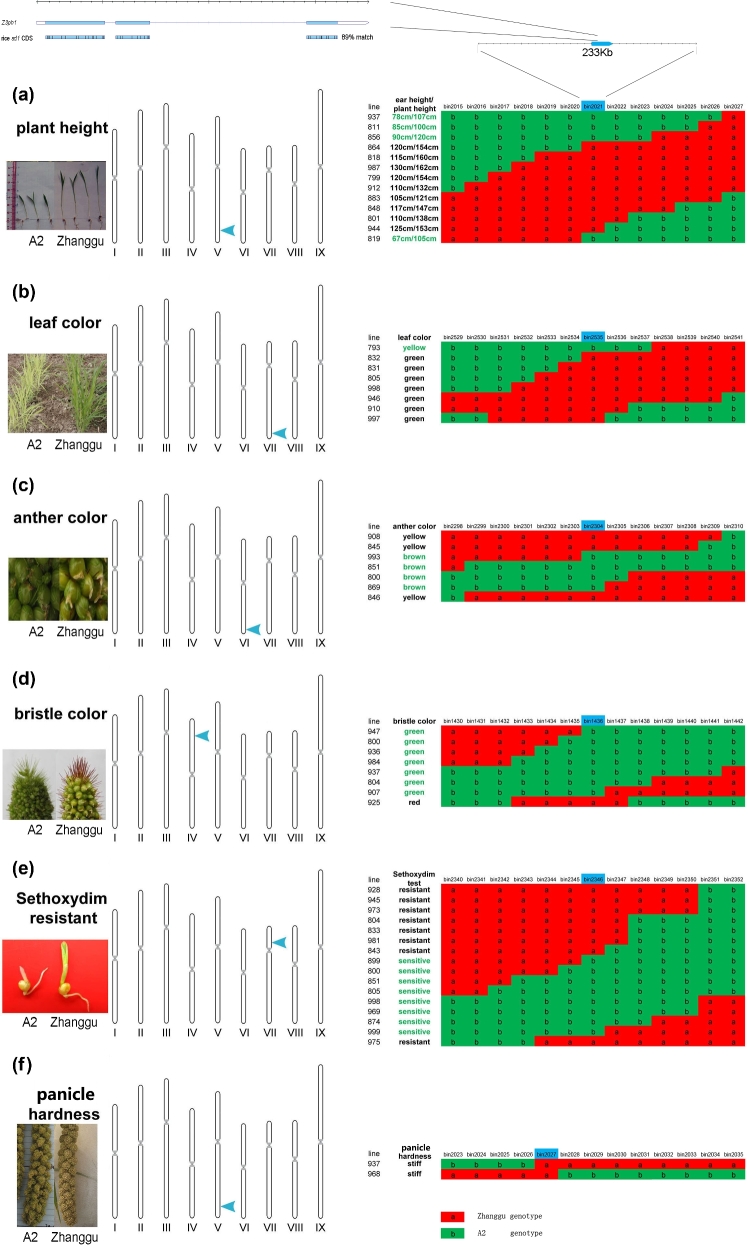
Gene mapping of the largest effect locus of plant height and five qualitative traits in foxtail millet. Genotype of recombination lines are shown in red and green block, “a” in red block means paternal genotype, “b” in green means maternal genotype. Phenotype of recombination lines are shown in the left of genotype blocks. (**a**) Gene mapping of the largest effect locus of plant height (paternal: tall – maternal: dwarf). (**b**) Gene mapping of the locus of leaf color (paternal: green – maternal: yellow). (**c**) Gene mapping of the locus of anther color (paternal: yellow – maternal: brown). (**d**) Gene mapping of the locus of bristle color (paternal: red – maternal: green). (**e**) Gene mapping of the locus of sethoxydim resistance (paternal: resistant – maternal: sensitive). (**f**) Gene mapping of the locus of tassel hardness (paternal: stiff – maternal: flexible).

All five qualitative traits show single gene control pattern (Fig. 5). According to the phenotypes of parents and F1, these five traits were controlled by dominant genes. Leaf color (green - yellow) was controlled by a locus *Z3lc* mapped onto the long arm of chromosome 7 (bin2535). Sethoxydim resistance (resistance - sensitive) was controlled by alocus*Z3sr* mapped onto the short arm of chromosome 7 (bin2346). Bristle color (red - green) was controlled by a locus *Z3bc* mapped onto the short arm of chromosome 4 (bin1436). Anther color (yellow - brown) was controlled by a loci *Z3ac* mapped onto the long arm of chromosome 6 (bin2304); panicle hardness (stiff - flexible) was controlled by a locus *Z3pah* mapped onto the short arm of chromosome 5 (bin2027).

**Figure 6.**
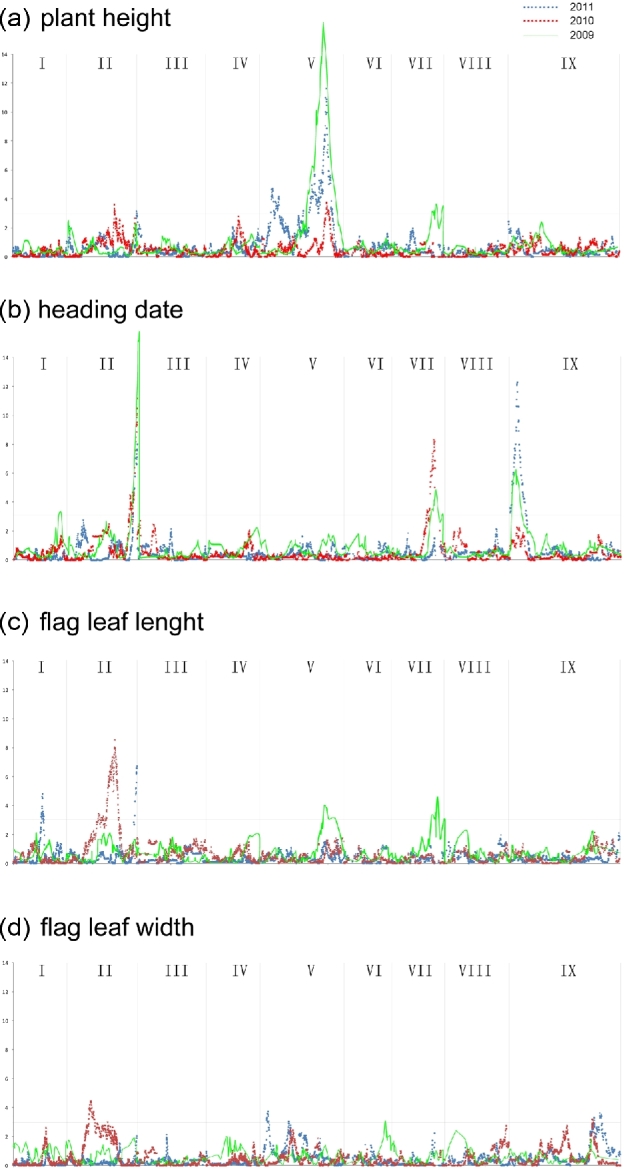
QTL analysis of five quantitative traits in foxtail millet. Peak signals of 3 years are shown in 3 colors. (**a**) QTL analysis of plant height. (**b**) QTL analysis of heading date. (**c**) QTL analysis of flag leaf length. (**d**) QTL analysis of flag leaf width. Lines in green means data collected in 2009, lines in red means data collected in 2010, lines in blue means data collected in 2011.

Using 184 RIL lines and a F2 population (the F2 population used to construct linkage map in 2009), we detected two QTL related to plant height (chr2, chr5, Fig. 6b). The largest effect locus (25.3% in 2011; 8.8% in 2010; 46.3% in 2009) was then mapped onto bin2021 (Fig. 5a). The candidate gene *Z3ph1* in bin2021 showed 89% identity to the known rice gibberellin-synthesis gene *sd1* [18] (Figs 5a and 6a), which indicated the plant height in foxtail millet might also be controlled by GA20ox. We also detected three quantitative trait loci (QTL) related to heading date (chr2, chr7, chr9). One locus was identical to the position of the leaf color control gene *Z3lc* (Fig. 6b). Flag leaf width and length were more complex than plant height and heading date. We detected five large effect QTLs related to flag leaf length, but no one showed repeated emergence in 3 years. QTLs related to flag leaf width were tiny and irregular, but one QTL located in chromosome 9 repeated in 3 years.

## Discussion

In this paper, the genome draft map, high density genetic linkage map and QTL mapping of several important agronomic traits were done using next generation sequencing [9] and Sanger sequencing [8]. We updated the millet draft genome to the millet fine map by adding unseated scaffolds (16 Mb data), which indicated that whole genome resequencing could provide more density markers for genetic map construction and could be very useful for improve genome quality. The Zhanggu and Yugu genome reference updated in this work will be helpful for foxtail millet genetic analysis in future. All the data are being made publicly available [19].

Grasses provide staple food for the vast majority of the world population. It can be divided into different tribes [20, 21]. Foxtail millet belongs to one of the tribe that contains many drought-resistant species, such as switchgrass (*Panicum virgatum*), pearl millet (*Pennisetum glaucum*), and prosomillet (*Panicum miliaceum*). Although foxtail millet is one of the most drought-tolerant crops, its planting area declined sharply in the last 30 years in China, mainly substituted by high-yield hybrid corn (http://www.nongtewang.org/grain/news/2016-01-07/50493.html). The most important reason was due to the relative low productivity and high labor cost of the traditional foxtail millet. Data showed that the foxtail millet also had distinct heterosis between different individuals, which was similar with the other grass crop rice [7, 22]. The high yield, herbicide resistance characteristics made hybrid foxtail millet suitable for large scale planting and industrialization [23–26].

The main influence factor of hybrid seed purity identification is the false hybrids from the male sterile parent self-cross. The yellow leaf of A2 can be used as indicator of false hybrids but it costs high labor and inefficiency. Breeding anti-herbicide lines could solve not only the problems of weed, but also problems of false-hybrid in seed production. Breeders found sethoxydim resistant in *Setaria viridis* and transferred it into foxtail millet. With our findings, breeders can take the method of MAS to transfer the herbicide-resistant into many lines that have wide male parents choose for hybrids. Meanwhile, the yellow leaf made the male sterile line A2 weak growth in seeding stage. After taking herbicide-resistant as an indicator of false-hybrid and the fine mapping of *Z3lc*, breeders can change the leaf color in short time. The trait of heading date is very important in foxtail millet genetic improvement. Three QTLs were found in this research and they could be used for breeding.

The transformation system of foxtail millet is difficult in that gene functional studies are hard to process. Our result of *Z3ph1* with 89% identity to the known rice gibberellin-synthesis gene *sd1* indicates that homology analysis may be taken between rice and foxtail millet.

## Availability of supporting data

The genome sequence and annotation data set of Zhanggu (second edition) has been deposited into NCBI (accession number: PRJNA73995). The genome sequence and annotation data set of Yugu (second edition) has been deposited into NCBI (accession number: PRJNA80183). The genome reference sequence and genotype of 184 RILs can be downloaded from the *GigaScience* GigaDB repository [19]. The details of 3437 bins can be found in Table S2.

## Supplementary data

Supplementary data are available at *GIGSCI* online.

**Table S1.** MSG sequencing and recombination events information.

**Table S2.** SNPs information generated from F2 population. The document includes the genotypes of samples. The missing genotype is markers as “-” specially. Format description (left to right): column 1: chromosome name; column 2: position; column 3: genotype of two parents and each F2 sample.

GIGA-D-16-00059_Original_Submission.pdf

GIGA-D-16-00059_Revision_1.pdf

Response_to_Reviewer_Comments_Original_Submission.pdf

Reviewer_1_Reort_(Original_Submission).pdf

Reviewer_2_Report_(Original_Submission).pdf

Reviewer_3_Report_(Original_Submission).pdf

Supplement Tables

## References

[1] Barton L, Newsome SD, Chen FH Agricultural origins and the isotopic identity of domestication in northern China. Proc Natl Acad Sci USA 2009;106:5523–8. 19307567 10.1073/pnas.0809960106 PMC2667055

[2] Zohary D, Hopf M Domestication of Plants in the Old World: The Origin and Spread of Cultivated Plants in West Asia, Europe, and the Nile Valley, 3rd edn Oxford: Oxford University Press, 2000.

[3] Bettinger RL, Barton L, Morgan C The origins of food production in north China: A different kind of agricultural revolution. Evol Anthropol 2010;19:9–21.

[4] Harlan JR Crops and Man. Madison, WI: American Society of Agronomy, 1975.

[5] Austin DF. Foxtail millets (*Setaria*: Poaceael) Abandoned food in two hemispheres. Econ Bot 2006;60:143–58.

[6] Dekker J. The foxtail (*Setaria*) species-group. Weed Sci 2003;51:641–56.

[7] Siles MM, Russell WK, Baltensperger DD Heterosis for grain yield and other agronomic traits in foxtail millet. Crop Sci 2004;44:1960–5.

[8] Bennetzen JL, Schmutz J, Wang H Reference genome sequence of the model plant setaria. Nature Biotechnol 2012;30:555–61. 22580951 10.1038/nbt.2196

[9] Zhang GY, Liu X, Quan ZW Genome sequence of foxtail millet (*Setaria italica*) provides insights into grass evolution and biofuel potential. Nature Biotechnol 2012;30:549–54. 22580950 10.1038/nbt.2195

[10] Murray MG, Thompson WF Rapid isolation of high molecular weight DNNucleic A. Acids Res 1980;8:4321–5. 10.1093/nar/8.19.4321 PMC324241 7433111

[11] Li H, Handsaker B, Wysoker A The sequence alignment/map format and SAMtools. Bioinformatics 2009;25:2078–9. 19505943 10.1093/bioinformatics/btp352 PMC2723002

[12] Huang X, Feng Q High-throughput genotyping by whole-genome resequencing. Genome Res 2009;19(6):1068–76. 19420380 10.1101/gr.089516.108 PMC2694477

[13] Duan M, Sun Z Genetic analysis of an elite super-hybrid rice parent using high-density SNP markers. Rice 2013;6(21), doi:10.1186/1939-8433-6-21. 10.1186/1939-8433-6-21 PMC4883714 24279921

[14] Wu Y, Bhat PR, Close TJ Efficient and accurate construction of genetic linkage maps from the minimum spanning tree of a graph. PLoS Genet 2008;4(10):e1000212 doi:10.1371/journal.pgen.1000212. 18846212 10.1371/journal.pgen.1000212 PMC2556103

[15] Van Ooijen JW, Kyazma BV MapQTL® 5, Software for the Mapping of Quantitative Trait Loci in Experimental Populations. Kyazma BV, Wageningen, Netherlands, 2004.

[16] Hirano R, Naito K, Fukunaga K Genetic structure of landraces in foxtail millet (*Setaria italica* (L.) P. Beauv.) revealed with transposon display and interpretation to crop evolution of foxtail millet. Genome 2011;54:498–506. 21623678 10.1139/g11-015

[17] Naciri Y, Darmency H, Belliard J Breeding strategy in foxtail millet, *Setaria italica* (L.P. Beauv.), following interspecific hybridization. Euphytica 1992;60:97–103.

[18] Sasaki A, Ashikari M, Ueguchi-tanaka M Green revolution: a mutant gibberellin-synthesis gene in rice. Nature 2002;416:701–2. 11961544 10.1038/416701a

[19] Ni X, Xia Q, Zhang H Gene mapping data of nine agronomic traits and genome assembly data of a foxtail millet RIL population GigaScience Database; 2016 http://dx.doi.org/10.5524/100213. 10.1093/gigascience/giw005 PMC5466707 28369461

[20] Doust AN, Kellogg EA, Devos KM Foxtail millet: a sequence-driven grass model system. Plant Physiol 2009;149:137–41. 19126705 10.1104/pp.108.129627 PMC2613750

[21] Lata C, Gupta S, Prasad M Foxtail millet: a model crop for genetic and genomic studies in bioenergy grasses. Crit Rev Biotechnol 2013;33(3):328–43. 22985089 10.3109/07388551.2012.716809

[22] Huang X, Wei X, Sang T, Zhao Q, Feng Q Genome-wide association studies of 14 agronomic traits in rice landraces. Nat Genet 2010;42:961–7. 20972439 10.1038/ng.695

[23] Doust AN, Devos KM, Gadberry MD Genetic control of branching in foxtail millet. Proc Natl Acad Sci USA 2004;101:9045–50. 15184666 10.1073/pnas.0402892101 PMC428470

[24] Jia GQ, Huang XH, Zhi H A haplotype map of genomic variations and genome-wide association studies of agronomic traits in foxtail millet (*Setaria italica*). Nat Genet 2013;45(8):957–61. 23793027 10.1038/ng.2673

[25] Jia XP, Shi YS, Song YC Development of EST-SSR in foxtail millet (*Setaria italica*). Genet Resour Crop Evol 2007;54:233–6.

[26] Wang CF, Jia GQ, Zhi H Genetic diversity and population structure of Chinese foxtail millet (*Setaria italica* (L.) Beauv.) landraces. G3 (Bethesda) 2012;2(7):769–77. 22870400 10.1534/g3.112.002907 PMC3385983

