# Supplementary material for: Updated foxtail millet genome assembly and gene mapping of nine key agronomic traits by resequencing a RIL population
Source: Gigascience. 2017 Jan 20;6(2):1–8. doi: 10.1093/gigascience/giw005 (PMC5466707; doi:10.1093/gigascience/giw005)

Manuscript Click here to download Manuscript Manucript-1020(1).doc

# Updated foxtail millet genome assembly and gene mapping of nine key agronomic traits by resequencing a RIL population~~Gene mapping of nine agronomic traits and genome assembly by resequencing a foxtail millet RIL population~~

Xuemei Ni[1,3,†], Qiuju Xia[1,3,†], Houbao Zhang[1,3], Shu Cheng[1,3], Hui Li[1,3], Guangyu Fan[2], Tao Guo[1,3], Ping Huang[1,3], Haitao Xiang[1,3], Qingchun Chen[1,3], Ning Li[1,3], Hongfeng Zou[1,3], Xuemei Cai[1,3], Xuejing Lei[1,3], Xiaoming Wang[2], Chengshu Zhou[1,3,4,5,6], Zhihai Zhao[2], Gengyun Zhang[1,3,4,5,6], Zhiwu Quan[1,3,4,5,6,*]

[†]These authors contributed equally to this work.

[1] BGI-Shenzhen, Shenzhen 518083, China.

[2] Institute of millet, Zhangjiakou Academy of Agricultural Science, Zhangjiakou 075000, China.

[3] State Key Laboratory of Agricultural Genomics, BGI-Shenzhen, Shenzhen 518083, China.

[4] Key Lab of Genomics ,Chinese Ministry of Agriculture, BGI-Shenzhen, Shenzhen 518083, China.[5] Guangdong Province key laboratory of crop germplasm research and application, BGI-Shenzhen, Shenzhen 518083, China.

[6] Shenzhen engineering laboratory of molecular design breeding, BGI-Shenzhen, Shenzhen 518083, China.

Correspondence should be addressed. E-mail: quanzhiwu@genomics.cn

## ABSTRACT

Foxtail millet (*Setaria italica*) provides food and fodder in semi-arid regions and infertile land. Resequencing of 184 foxtail millet recombinant inbred lines (RILs) was carried out to aid essential research on foxtail millet improvement. A total of 483,414 SNPs were determined. Bin maps were constructed based on the RILs' recombination data. Based on the high density bin map, we updated Zhanggu reference with 416Mb after adding 16 Mb unanchored scaffolds and Yugu reference with some assembly errors correction and 3158 gaps filled.

Commented [SE1]: Undeleted some of this as it no longer made sense.

~~By anchoring some unseated scaffolds and filling gaps, we update two original millet reference genomes Zhanggu and Yugu to produce second editions. Gene~~ QTL mapping of nine agronomic traits were done based on this RIL population, five of which were controlled by a single gene. Meanwhile, two QTLs were found for plant height and a candidate gene showed 89% identity to the known rice gibberellin-synthesis gene *sd1*. Three QTLs were found for the trait of heading date. The whole genome resequencing and QTL mapping provided important tools for foxtail millet research and breeding. Resequencing of the RILs could also provide an effective way for high quality~~quantity~~ genome assembly and gene identification.


## INTRODUCTION

Foxtail millet (*Setaria italica*) was an ancient cultivated crop domesticated in China more than 8,700 years ago (ZOHARY and HOPF 2000; BARTON *et al.* 2009). It provided the most important food and forage for the Yellow River valley in ancient China and is still as an essential food source in semi-arid areas (HARLAN 1975; BETTINGER *et al.* 2010). Although foxtail millet is one of the most drought-tolerant~~resistant~~ crops, the low productivity of unimproved foxtail millet has limited its application in agriculture. In the past decade, high-yield hybrids with herbicide-resistant ~~herbicide resistant, high yield hybrid millet~~ which can dramatically improve crop production and decrease labor cost were inbred, indicating that foxtail millet had~~s~~ the potential for becoming a high yield crop through the use of genetic tools (DEKKER 2003; SILES *et al.* 2004;

Austin 2006).

Foxtail m~~M~~illet genome *de novo* sequencing was initially finished in 2012 (Zhang *et al.* 2012; Bennetzen *et al.* 2012), here we carried out resequencing of foxtail millet RIL population and construction of the high resolution bin map. A high accuracy Zhanggu millet genome reference (anchoring 96% scaffold sequence) was constructed using the high resolution bin map based on the previous Zhanggu draft genome~~based on bin map and Zhanggu draft genome~~ (Zhang *et al.* 2012). Additionally, this bin map was also used to check the assembly error in Yugu genome reference (Bennetzen *et al.* 2012) and~~, a Yugu genome reference~~ second edition (anchoring 99.7% scaffold sequence) was done after assembly error correction and gap filling. Nine agronomic traits were phenotyped in 2010 and 2011, respectively.~~The RIL population was phenotype appraisal for nine agronomic traits in 2010 and 2011, respectively.~~ ~~Gene mapping and~~ QTL analysis was done based on genotype and phenotype data~~e~~ ~~of these RILs~~, and some loci were mapped at a 130 Kb region~~zone~~ using these 184 RILs, indicating that resequencing of foxtail millet RIL population could provide an effective approach for high quality~~quantity~~ genome assembly and gene mapping. The genome reference and SNP markers will become an important tool in foxtail millet molecular breeding, and the loci related to the nine agronomic traits will provide pivotal information to ~~millet~~ breeders.

## MATERIALS AND METHODS

No specific permissions were required for the described field studies. The location i is not privately owned or protected, and the field studies did not involve endangered o r protected species.

**Sampling and RIL construction**

Zhanggu was selected as the male parent line and A2, a popular male sterile line which was used widely for hybrid breeding, was selected as the –female parent line. ~~male sterile line~~. F1 was constructed from a cross between Zhanggu and A2, RILs was developed ~~by~~ using single seed descent strategy. Segregation population was grown

three generations per year in New Village, Jiyang Town, Sanya City, Hainan province (Coordinates: 109°35′E/18°17′N) (November-January; January-April) and Erliban Village, Shalingzi Town, Xuanhua County, Zhangjiakou City, Hebei~~Hainan~~ province (Coordinates: 114°54′E/40°40′N) (May-October).

**Phenotyping**

Nine agronomic traits contained flag leaf length (FLL), leaf color (LC), bristle color (BC), anther color (AC), plant height (PH), ear height (EH), heading date (HD), panicle hardness (PAH) and sehtoxydim resistant (SR).

The male parent Zhanggu had green leaf, red bristle, yellow anther with sethoxydim-resistant, while the A2 had yellow leaf, green bristle, brown anther with sethoxydim-sensitive. F1 had green leaf, red bristle, brown anther with sethoxydim-resistant.

All the color traits were characterized according to the RILs' appearance of different development periods. Leaf color was observed and recorded as yellow or green 15-days after sowing. The red or green bristle color was collected 10-days after heading and anther color was recorded as yellow or brown during flowing period.

Heading date was recorded as the number of days from sowing to heading. Flag leaf length and width were measured at the maximal values for each flag leaf using a ruler. Plant height was measured the distance between ~~tassel~~ panicle terminal and ground. Additionally, ear height was measured the distance between flag leaf ear and ground. ~~Heading date was recorded as the number of days from sowing to heading.~~ Panicle hardness was observed and recorded as stiff or flexible 30 days after flowering.

Sethoxydim resistant data was collected after 3-days ~~by~~ seeding on Sethoxydim medium (50mg/L), normal growing recording resistant and wilting or dead recording sensitive. ~~. Leaf color was observed and record 15days after sowing, leaf color can divide into yellow and green. Bristle color was observed and record 10days after heading, bristle color can divide into red and green. Anther color was observed and record when flower, anther color can divide into yellow and brown. Tassel hardness was observed and record 30 days after flowering, tassel can divide into stiff and~~

flexible.

**DNA isolation and genome sequencing**

Total genomic DNA was extracted from young leaf tissues of F10 using the CTAB method (Murray and Thompson 1980). DNA was quantified using 1% agarose gel electrophoresis and Qubit Fluorometer. 500-bp pair-end libraries wereas constructed under the standard protocol provided by Illumina (San Diego, USA). The sequencing was performed using Hiseq-2000 for pair-end 50-cycle sequencing according to the manufacturer's standard protocol. Low quality reads, reads with adaptor sequences, duplicated reads were filtered and the remained high quality data was used in SNP calling.

**Sequence alignment, genotyping and recombination breakpoint determination**

Reads of all samplesA2 wereas mappedmapping onto Zhanggu initial genome reference (containing 9 pseudo chromosomes and unmapped scaffolds)scaffold by using SOAP2 (Li *et al.* 2009) (vision 2.20)., The input data for SNP calling was prepared by conducted by using SAMtools (Li *et al.* 2009) (version 0.1.8) and then SNP calling was conducted by realSFS (version 0.983), based on the Bayesian estimation of site frequency at each site. The SNPs for further analysis were selected by the following criteria: the different alleles between two parents with missing data less than 60%. Positions of these SNPs were marked for RIL SNP calling. After mapping the reads of each RIL back to the scaffolds of Zhanggu, SAMtools (Li *et al.* 2009) (version 0.1.8) and realSFS (version 0.983) were used to identify SNPs in each RIL.Then A a sliding window approach was used to evaluate 15 consecutive SNPs for genotype calling and continued the process as the window slide base-by-base (Huang *et al.* 2009Wu *et al.* 2008). This approach was adopted in several researches and we followed their methods for genotyping identification (Huang *et al.* 2009; Duan *et al.* 2013; Wang *et al.* 2015). The window with a Zhanggu:A2 SNPs ratio of 11:4 or higher was called Zhanggu genotype, 4:11 or lower called A2 genotype, SNPs ratio between 11:4 and 4:11 was called heterozygous. The breakpoint was determined at the boundary of the Zhanggu, A2 and heterozygous.

**Bin map and chromosome construction**

All breakpoints were aligned along the Zhanggu initial chromosomes sorted from the upper end to bottom end with 20Kb minimal intervals. All SNP data of the 184 RILs were aligned to a matrixs, the minimal interval of two recombination positions was set as 50kb. Adjacent intervals with the same genotype across the 184 RILs were defined as a single recombination bin (WU *et al.* 2008). 3437 The recombination bins were serving as 3437 genetic markers and, the linkage map was constructed using MSTMap with Kosambi's mapping function (http://alumni.cs.ucr.edu/~yonghui/mstmap.html WU *et al.* 2008). Then the Zhanggu genome was updated New chromosomes were constructed based on bin map.

Commented [SE2]: Needs to be cited in the references

**Gene mapping and QTL mapping**

Phenotype of each RIL and genotype of each bin was collected for gene mapping and QTL analysis. QTLs were identified using composite interval mapping performed in the software package MapQTL 5 (VAN OOIJEN and KYAZMA 2004).The likelihood ratio statistic was computed every bin, QTL were called for LOD values higher than 3.0.

**Yugu genome construction and gap filling**

Reads of A2 and RILs were mapping to the chromosomes and scaffolds of Yugu (BENNETZEN *et al.* 2012). The SNP calling procedure was the same as above method. , SNPs between RILs and Yugu were identified using SOAPsnp (LI *et al.* 2009) (Version 1.02). The differences were that we couldn't identify the Zhanggu allele directly. So we imputated the male parent allele according the population SNPs, that is, SNP identical to A2 was considered as A2 allele and , SNP contrary to A2 was considered as Zhanggu allele. Bin map was constructed using the same strategy as before, bins with abnormal linkage were move to proper position according to the bin map. Gaps with two flank sequence werewas mappeding to Zhanggu genome., Aa gap was filled withby Zhanggu sequence only when both flank sequences wereas matched to Zhanggu sequence. Zhanggu sequence filled in Yugu gap was shown in lowercase (Fig. 2).

## RESULTS

~~Two foxtail millet inbred line were selected as the parents: Zhanggu as the male parent line and A2 as the male sterile line. RILs were developed by hybridization between Zhanggu and A2 followed by self fertilization to F10. Nine agronomic traits were measured for each RIL in 2010 and 2011, respectively.~~

~~DNAs were isolated from each RIL's young leaves using CTAB method (Murray and Thompson 1980). 500 bp pair end libraries were constructed according to the standard protocol provide by Illumina (San Diego, USA). The sequencing was performed using Hiseq 2000 for pair end 50 cycle sequencing according to the manufacturer's standard protocol. Low quality reads, adaptor sequences and duplicated reads were filtered, the remaining high quality data was used for SNP calling.~~ A total of~~In total~~ 140~~-~~Gb clean data was generated, which given a 2X depth~~coverage~~ of each RIL on average. ~~Genetic variations were detected by comparision between the genome sequence of Zhanggu and resequencing (~10X) of A2 (Zhang *et al.* 2012). Reads of A2 were mapped onto Zhanggu scaffolds using SOAP2 (Li *et al.* 2009) (version 2.20), SNP calling was conducted using SAMtools (Li *et al.* 2009) (version 0.1.8) and realSFS (version 0.983). Positions of these SNPs were marked for RIL SNP calling. After mapping the reads of each RIL back to the scaffolds of Zhanggu, SNPs in the marked position were selected for genotyping.~~ A total of 483,414 SNPs were detected, given an average density of 1.2 SNPs per Kb for the RILs (Table 1, Fig. 1).

~~To avoid potential sources of sequence errors and mapping errors, we chose a sliding window approach to evaluate 15 consecutive SNPs for genotype calling and continued the process as the window slide base by base (Wu *et al.* 2008). To improve the quality of millet draft genome and conduct genetic analyses, a bin map was constructed based on the recombination data (Wu *et al.* 2008). All SNP data of the 184 RILs were aligned to a matrixs, the minimal interval of two recombination positions was set as 10kb. Adjacent intervals with the same genotype across the 184 RILs were defined as a single recombination bin (Wu *et al.* 2008).~~ We identified~~y~~

3437 recombination bins in the 184 RILs, the physical length of the recombination bins ranging from ~~1~~ 20kb to 12Mb, given an average length of 121Kb (Table S1). ~~3437 single recombination bin were serving as 3437 markers, linkage map was constructed using MSTmap (Van Ooijen and Kyazma 2004).~~ 9 linkage groups, with a total genetic distance of 1927.8 cM (Table 1, Fig 2), were constructed ~~of the foxtail millet genome~~. The interval between these binsranged~~ing~~ from 0.1 cM to 13.8 cM, average at 0.56 cM. ~~New chromosomes~~ Chromosomes were updated ~~constructed~~ based on the bin map and scaffolds of Zhanggu, and the second edition Zhanggu reference genome was generated (416Mb) after adding unanchored scaffolds (16Mb) (Table 2, Fig. 4). ~~Reads of RILs were mapping to the chromosomes and scaffolds of Yugu to construct bin map,~~ The assembly errors of Yugu genome reference ~~was~~ were revised based on the bin map constructed by alignment on Yugu genome . 3158 gaps ~~was~~ were filled by ~~ZhangGu~~ Zhanggu sequences using sequence homology BLAST~~,~~ . ~~the~~ The second edition ~~YuGu~~ Yugu reference genome was constructed after assembly error correction and gap filling (Fig. 3).

To identify agronomic traits related loci which were important in foxtail millet, ~~gene mapping and~~ QTL analysis was done based on this RIL population (Wu *et al.* 2008; Hirano *et al.* 2011). Nine agronomic traits, which can be divided into two categories: qualitative traits (Sethoxydim resistance, leaf color, bristle color, anther color, ~~tassel~~ panicle hardness) and quantitative traits (plant height, heading date, flag leaf width, flag leaf length)~~, were measured with replications~~ (NACIRI et al. 1992).

All five qualitative traits show single gene control pattern (Fig. 5). According to the phenotypes of parents and F1, these five traits were controlled by dominant genes. Leaf color (green - yellow) was controlled by a ~~dominant~~ locus~~gene~~ *Z3lc* mapped onto the long arm of chromosome 7 (bin2535~~);~~ ). Sethoxydim resistance (resistance - sensitive) was controlled by ~~a~~a ~~dominant gene~~locus~~-~~ *Z3sr* mapped onto the short arm of chromosome 7 (bin2346~~);~~ ). ~~bristle~~ Bristle color (red - green) was controlled by a ~~dominant~~ locus~~gene~~ *Z3bc* mapped onto the short arm of chromosome 4 (bin1436~~);~~ ). ~~anther~~ Anther color (yellow - brown) was controlled by a ~~dominant~~ loci~~gene~~ *Z3ac*

mapped onto the long arm of chromosome 6 (bin2304); ~~tassel~~ panicle hardness (stiff - flexible) was controlled by a ~~dominant gene~~ locus ~~*Z3th*~~ *Z3pah* mapped onto the short arm of chromosome 5 (bin2027).

Using 184 RIL lines and a F2 population (the F2 population used to construct linkage map in 2009), we detected two quantitative trait loci (QTL) related to plant height (chr2, chr5, Fig. 6 b). The largest effect locus (25.3% in 2011; 8.8% in 2010; 46.3% in 2009) was then mapped onto the bin2021 (Fig. 5 a). The candidate gene *Z3ph1* in bin2021 shown 89% identity to the known rice gibberellin-synthesis gene *sd1* (SASAKI *et al.* 2002) (Fig. 5 a, Fig. 6 a), which indicated the plant height in foxtail millet might~~ay~~ also be controlled by GA20ox. We also detected three quantitative trait loci (QTL) related to heading date (chr2, chr7, chr9). One locus ~~which is~~ was identical to the position of the leaf color control gene *Z3lc*~~, which indicated that the leaf color also affect the heading date~~ (Fig. 6 b). Flag leaf width and length ~~are~~ were more complex than plant height and heading date. We detected five large effect QTLs related to flag leaf length, but no one showed repeated emergence in three years. QTLs related to flag leaf width ~~are~~ were tiny and irregular, but one QTL located in chromosome 9 repeated in three years.

# DISCUSSION~~S~~

In this paper, the genome draft map, high density genetic linkage map and QTL mapping of several important agronomic traits were done using next generation sequencing (ZHANG *et al.* 2012) and Sanger sequencing (BENNETZEN *et al.* 2012) We updated the millet draft genome to the millet fine map by adding unseated scaffolds (16Mb data), which indicated that whole genome resequencing could provide more density markers for genetic map construction and could be very useful for improve genome quality. The Zhanggu and Yugu genome reference updated in this work will be helpful for foxtail millet genetic analysis in future. All the data ~~can~~is being made publicly available (NI *et al.* 2016).

Grasses provide staple food for the vast majority of the world population. It can be

divided into different tribes (Doust *et al*. 2009; Lata *et al*. 2013). Foxtail millet belongs to one of the tribe which contains many drought-resistant species, such as switchgrass (*Panicum virgatum*), pearl millet (*Pennisetum glaucum*), prosomillet (*Panicum miliaceum*). Although foxtail millet ~~was~~ is one of the most drought-tolerant crops, its planting area declined sharply in the last 30 years in China, mainly substituted by high yield hybrid corn (http://www.nongtewang.org/grain/news/2016-01-07/50493.html)~~it was still substituted by high yield bybrid corn~~. The most important reason was due to the relative low productivity and high labor cost of the traditional foxtail millet. Data showed that the foxtail millet also had distinct heterosis between different individuals, which was similar with the other grass crop rice (Siles *et al*. 2004; Huang *et al*. 2010). The high yield, herbicide resistance characteristics made hybrid foxtail millet suitable for large scale planting and ~~industrilization~~industrialization (Doust *et al*. 2004; Jia *et al*. 2007; Wang *et al*. 2012; Jia *et al*. 2012)~~.~~.

Commented [SE3]: Need to cite in references

The main influence factor of hybrid seed purity identification is the false hybrids from the male sterile parent self-cross. The yellow leaf of A2 can be used as indicator of false hybrids but it costs high labor and inefficiency. Breeding anti-herbicide lines could solve not only the problems of weed, but also problems of false-hybrid in seed production. Breeders found sethoxydim resistant in *Setaria viridis* and transferred it into foxtail millet. With our findings, breeders can take the method of MAS to transfer the herbicide-resistant into many lines that have wide male parents choose for hybrids. Meanwhile, the yellow leaf made the male sterile line A2 weak growth in seeding stage. After taking herbicide-resistant as an indicator of false-hybrid and the fine mapping of *Z3lc*, breeders can change the leaf color in short time. The trait of heading date is very important in foxtail millet genetic improvement. Three QTLs were found in this research and they could be used for breeding.

The transformation system of foxtail millet is difficult that gene functional studies are hard to process. According to our result that *Z3ph1* with 89% identity to the known rice gibberellin-synthesis gene *sd1*, it indicates that homology analysis may be taken between rice and foxtail millet.

~~In this paper, the genome draft map, high density genetic linkage map and QTL mapping of several important agronomic trait were done using next generation sequencing (ZHANG *et al*. 2012) and Sanger sequencing (BENNETZEN *et al*. 2012) method until 2012.~~

**Formatted:** Indent: First line: 0 ch

~~We developed the bin map and high density SNP markers, of which the data has been made publicly available. We also provided gene mapping and QTL mapping of nine important agronomic traits, the peak signals at nine loci will provide an important tool to foxtail millet breeding. What is more, we update the millet draft genome to the millet fine map by adding unseated scaffolds (16Mb data). The Zhanggu and Yugu genome reference updated in this work will be helpful for millet resequencing and GWAS analysis in future. Our study also indicates that resequencing of RILs could provide an effective approach for high quantity genome assembly and gene mapping.~~


## ACKNOWLEDGMENTS

We are grateful to all participants of the Agriculture department at BGI. The work was supported by the National Key Technology R&D Program (2015BAD02B01-7), the Technology Innovation Program Support by Shenzhen Municipal Government (JSGG20130918102805062 and CXZZ2015033017181006), the Basic Research Program Support by Shenzhen Municipal Government (JCYJ20150831201123287 and JCYJ20120618172523025). The funders had no role in study design, data collection and analysis, decision to publish, or preparation of the manuscript.

## Availability of supporting data

The genome sequence and annotation data set of Zhanggu (second edition) has been deposited into NCBI (accession number: PRJNA73995). The genome sequence and annotation data set of Yugu (second edition) has been deposited into NCBI (accession number: PRJNA80183). The genome reference sequence and genotype of 184 RILs can be downloaded from the *GigaScience* GigaDB repository (**NI *et al.* 2016**). The details of 3437 bins can be found in Table S2.

## LITERATURE CITED


AUSTIN, D. F., 2006 Foxtail millets (*Setaria*: Poaceael) Abandoned food in two hemispheres. Economic Botany 60: 143-158.

BARTON, L., S. D. NEWSOME, F. H. CHEN, H. WANG, T. P. GUILDERSON, R. L. BETTINGER, 2009 Agricultural origins and the isotopic identity of domestication in northern China. Proc Natl Acad Sci USA 106: 5523-5528.

BENNETZEN, J. L., J. SCHMUTZ, H. WANG, R. PERCIFIELD, J. HAWKINS *et al.*, 2012 Reference genome sequence of the model plant setaria. Nature Biotechnology 30: 555-561.

BETTINGER, R. L., L. BARTON, C. MORGAN, 2010 The origins of food production in north China: A different kind of agricultural revolution. Evol Anthropol 19: 9-21.

DEKKER, J., 2003 The foxtail (*Setaria*) species-group. Weed Sci 51: 641-656.

DOUST, A. N., E. A. KELLOGG, K. M. DEVOS, J. L. BENNETZEN, 2009 Foxtail millet: A sequence-driven grass model system. Plant Physiol 149: 137-141.

DOUST, A. N., K. M. DEVOS, M. D. GADBERRY, M. D. GALE, E. A. KELLOGG, 2004 Genetic control of branching in foxtail millet. Proc Natl Acad Sci USA 101: 9045-9050.

DOUST, A. N., K. M. DEVOS, M. D. GADBERRY, M. D. GALE, E. A. KELLOGG, 2005 The genetic basis for inflorescence variation between foxtail and green millet

(*poaceae*). Genetics 169: 1659-1672.

Duan M, Sun Z, *et al.*, 2013 Genetic analysis of an elite super-hybrid rice parent using high-density SNP markers. Rice 6(21): doi:10.1186/1939-8433-6-21.

Harlan, J. R., 1975 Crops and Man. American Society of Agronomy, Madison, Wisconsin.

Hirano, R., K. Naito, K. Fukunaga, K. N. Watanabe, R. Ohsawa, M. Kawase, 2011 Genetic structure of landraces in foxtail millet (*Setaria italica* (L.) P. Beauv.) revealed with transposon display and interpretation to crop evolution of foxtail millet. Genome 54: 498-506.

Huang X, Feng Q, *et al.*, 2009 High-throughput genotyping by whole-genome resequencing. Genome Research 19(6): 1068-1076.

Huang, X., X. Wei, T. Sang, Q. Zhao, Q. Feng *et al.*, (2010) Genome-wide association studies of 14 agronomic traits in rice landraces. Nat Genet 42: 961-967.

Jia, G. Q., X. H. Huang, H. Zhi, Y. Zhao, Q. Zhao *et al.*, 2013 A haplotype map of genomic variations and genome-wide association studies of agronomic traits in foxtail millet (*Setaria italica*). Nature Genetics 45(8): 957-961.

Jia, X. P., Y. S. Shi , Y. C. Song, G. Y. Wang, T. Y. Wang, Y. Li, 2007 Development of EST-SSR in foxtail millet (*Setaria italica*). Genet Resour Crop Evol 54: 233-236.

Lata, C., S. Gupta, M. Prasad, 2013 Foxtail millet: a model crop for genetic and genomic studies in bioenergy grasses. Crit Rev Biotechnol 33(3): 328-343.

Li, H., B. Handsaker, A. Wysoker, T. Fennell, J. Ruan *et al.*, 2009 The Sequence Alignment/Map format and SAMtools. Bioinformatics 25: 2078-2079.

Li, R., C. Yu, Y. Li, T. W. Lam, S. M. Yiu *et al.*, 2009 SOAP2: an improved ultrafast tool for short read alignment. Bioinformatics 25: 1966 -1967.

Murray, M. G., W. F. Thompson, 1980 Rapid isolation of high molecular weight DNA. Nucleic Acids Res 8: 4321-4325.

Naciri, Y., H. Darmency, J. Belliard, F. Dessaint, J. Pernès, 1992 Breeding strategy in foxtail millet, *Setaria italica* (L.P.Beauv.), following interspecific

hybridization. Euphytica 60: 97-103.
Ni, X; Xia, Q; Zhang, H; Guo, T; Fan, G; Wang, R; Cai, Y; Huang, P; Xiang, H; Liu, C; Chen, Q; Li, N; Zou, H; Cai, X; Lei, X; Zhang, R; Wang, X; Zhou, C; Zhao, Z; Zhang, G; Quan, Z (2016): Gene mapping data of nine agronomic traits and genome assembly data of a foxtail millet RIL population GigaScience Database. http://dx.doi.org/10.5524/100213

Sasaki, A., M. Ashikari, M. Ueguchi-tanaka, H. Itoh, A. Nishimura *et al.*, 2002 Green revolution: a mutant gibberellin-synthesis gene in rice. Nature 416: 701-702.

Siles, M. M., W. K. Russell, D. D. Baltensperger, L. A. Nelson, B. Johnson *et al.*, 2004 Heterosis for Grain Yield and Other Agronomic Traits in Foxtail Millet. Crop Sci 44: 1960-1965.

Van ooijen, J. W., B. V. Kyazma, 2004 MapQTL® 5, software for the mapping of quantitative trait loci in experimental populations. Wageningen.

Wang, C. F., G. Q. Jia, H. Zhi, Z. G. Niu, Y. Chai *et al.*, 2012 Genetic diversity and population structure of Chinese foxtail millet (*Setaria italica* (L.) Beauv.) landraces. G3 (*Bethesda*) 2(7): 769-777.

WANG, C. F., J. F. CHEN, H. ZHI, L. YANG , W. LI *et al.*, 2010 Population genetics of foxtail millet and its wild ancestor. BMC Genet 11: 90.

Wang L, Xia Q, Zhang Y, Quan Z, Zhang X *et al.*, 2016 Updated sesame genome assembly and fine mapping of plant height and seed coat color QTLs using a new high-density genetic map. BMC Genomics 17:31.

Wu, Y., P. R. Bhat, T. J. Close, S. Lonardi, 2008 Efficient and Accurate Construction of Genetic Linkage Maps from the Minimum Spanning Tree of a Graph. PLoS Genet 4(10): e1000212. doi:10.1371/journal.pgen.1000212.

Zhang, G. Y., X. Liu, Z. W. Quan, S. F. Cheng, X. Xu *et al.*, 2012 Genome sequence of foxtail millet (*Setaria italica*) provides insights into grass evolution and biofuel potential. Nature Biotechnology 30: 549-554.

Zohary, D., M. Hopf, 2000 Domestication of plants in the old world: the origin and spread of cultivated plants in West Asia, Europe, and the Nile Valley. 3rd edn.

Oxford University Press.

## FIGURE LEGENDS

**Figure 1** Distribution of 483,414 SNPs between Zhanggu and A2.

**Figure 2** Genetic distance *vs.* physical distance. Genetic position of the 3437 bins is plotted against the corresponding physical position. Regions with low ratio of genetic distance to physical distance show heterochromatin regions

**Figure 3** Gap filling in Yugu chromosome. Gap was filled by Zhanggu sequence when both flank sequence was match to Zhanggu chromosome. (**a**) Gap was caused by low coverage, both flank sequence was match to Zhanggu, gap was fill by lowercase Zhanggu sequence. (**b**) Gap was caused by ploy G sequence, Sanger sequencing can't step over the ploy G.

**Figure 4** Genomic landscape of the Zhanggu chromosomes (second edition). Major DNA components are categorized into genes (brown), DNA transposons (yellow), Copia-like retrotransposons (dark blue), Gypsy-like retrotransposons (light blue), with respective DNA contents of 19%, 13%, 10% and 21% of the genome sequence. Categories were determined for 1-Mb windows with a 0.2-Mb shift. Recombination ratio was shown in blue bars, ranging from 0 cM/Mb to 30 cM/Mb.

**Figure 5** Gene mapping of the largest effect locus of plant height and five qualitative traits in foxtail millet. Genotype of recombination lines are shown in red and green block, "a" in red block means paternal genotype, "b" in green means maternal genotype. Phenotype of recombination lines are shown in the left of genotype blocks. (**a**) Gene mapping of the largest effect locus of plant height (paternal: tall – maternal: dwarf). (**b**) Gene mapping of the locus of leaf color (paternal: green – maternal: yellow). (**c**) Gene mapping of the locus of anther color (paternal: yellow – maternal:

brown). (**d**) Gene mapping of the locus of bristle color (paternal: red – maternal: green). (**e**) Gene mapping of the locus of Sethoxydim resistance (paternal: resistant – maternal: sensitive). (**f**) Gene mapping of the locus of tassel hardness (paternal: stiff – maternal: flexible).

**Figure 6** QTL analysis of five quantitative traits in foxtail millet. Peak signals of three years date are shown in three colors. (**a**) QTL analysis of plant height. (**b**) QTL analysis of heading date. (**c**) QTL analysis of flag leaf length. (**d**) QTL analysis of flag leaf width. Lines in green means data collected in 2009, lines in red means data collected in 2010, lines in blue means data collected in 2011.

## Supporting information

**Table S1 MSG sequencing and Recombination events information**

**(XLS)**

**Table S2 SNPs information generated from F2 population.** The document includes the genotypes of samples. The missing genotype is markers as "-" specially. Format description (left to right): Column1: Chromosome name. Column2: Position. Column3: Genotype of two parents and each F2 sample.

**(XLS)**

## Tables

**Table 1 Summary of the bins and SNP distribution in Zhanggu**

| Chromosome | Length(bp) | Bin num | Linkage(cM) | SNP num | SNP density(/Kb) |
|---|---|---|---|---|---|
| chr1 | 44603498 | 408 | 167.117 | 42624 | 0.955 |
| chr2 | 51761675 | 491 | 224.647 | 89674 | 1.732 |
| chr3 | 54090027 | 396 | 221.738 | 81236 | 1.502 |
| chr4 | 43349090 | 348 | 168.232 | 27932 | 0.644 |

| | | | | | |
|---|---|---|---|---|---|
| chr5 | 49560508 | 480 | 267.52 | 60305 | 1.217 |
| chr6 | 36928436 | 203 | 152.1 | 30946 | 0.838 |
| chr7 | 37743793 | 242 | 168.48 | 56045 | 1.485 |
| chr8 | 38066565 | 283 | 205.431 | 58149 | 1.528 |
| chr9 | 59875680 | 586 | 352.58 | 36503 | 0.609 |
| Total | 415979272 | 3437 | 1927.845 | 483414 | 1.162 |

**Table 2 Summary of Zhanggu second edition and Yugu second edition**

| Strain | Chromosome length(bp) | Gap lenght(bp) | Gap number | Gap ratio | Filled gap number |
|---|---|---|---|---|---|
| Zhangg~~G~~u | 399,854,594 | 26,817,695 | 31942 | 6.7% | / |
| ~~ZhangGu$^{2th}$~~ Zhanggu$^{2th}$ | 415,979,272 | 28,962,873 | 34452 | 7.0% | / |
| ~~YuGu~~Yugu | 401,300,876 | 4,616,102 | 6171 | 1.2% | / |
| ~~YuGu$^{2th}$~~Yugu$^{2th}$ | 402,520,233 | 2,175,332 | 3297 | 0.5% | 2874 |

 Click here to download Figure Fig 1.tif

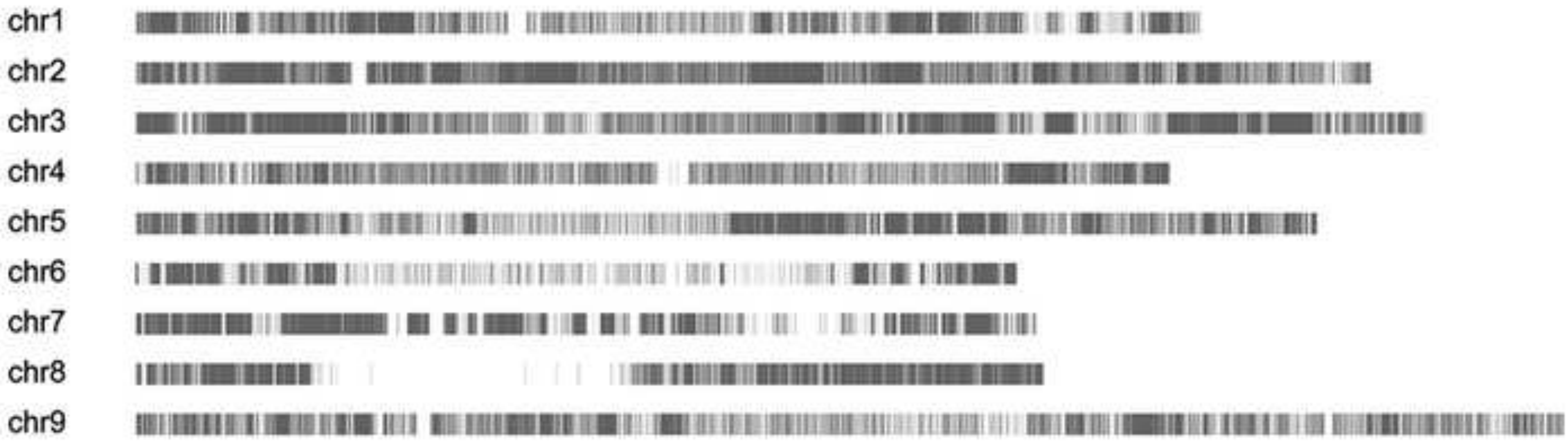

Figure2

Click here to download Figure Fig 2.tif

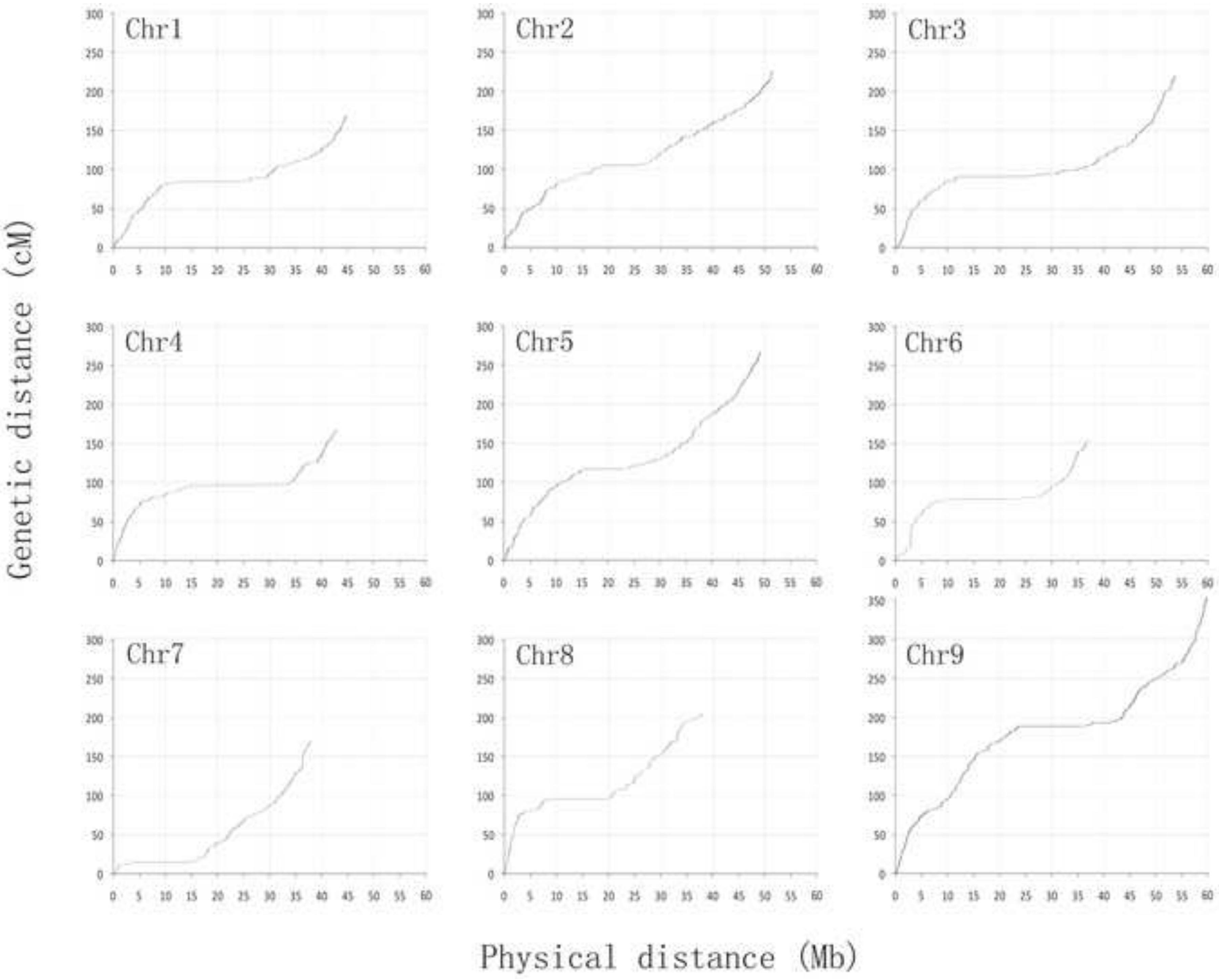

Figure3

Click here to download Figure Fig 3.tif

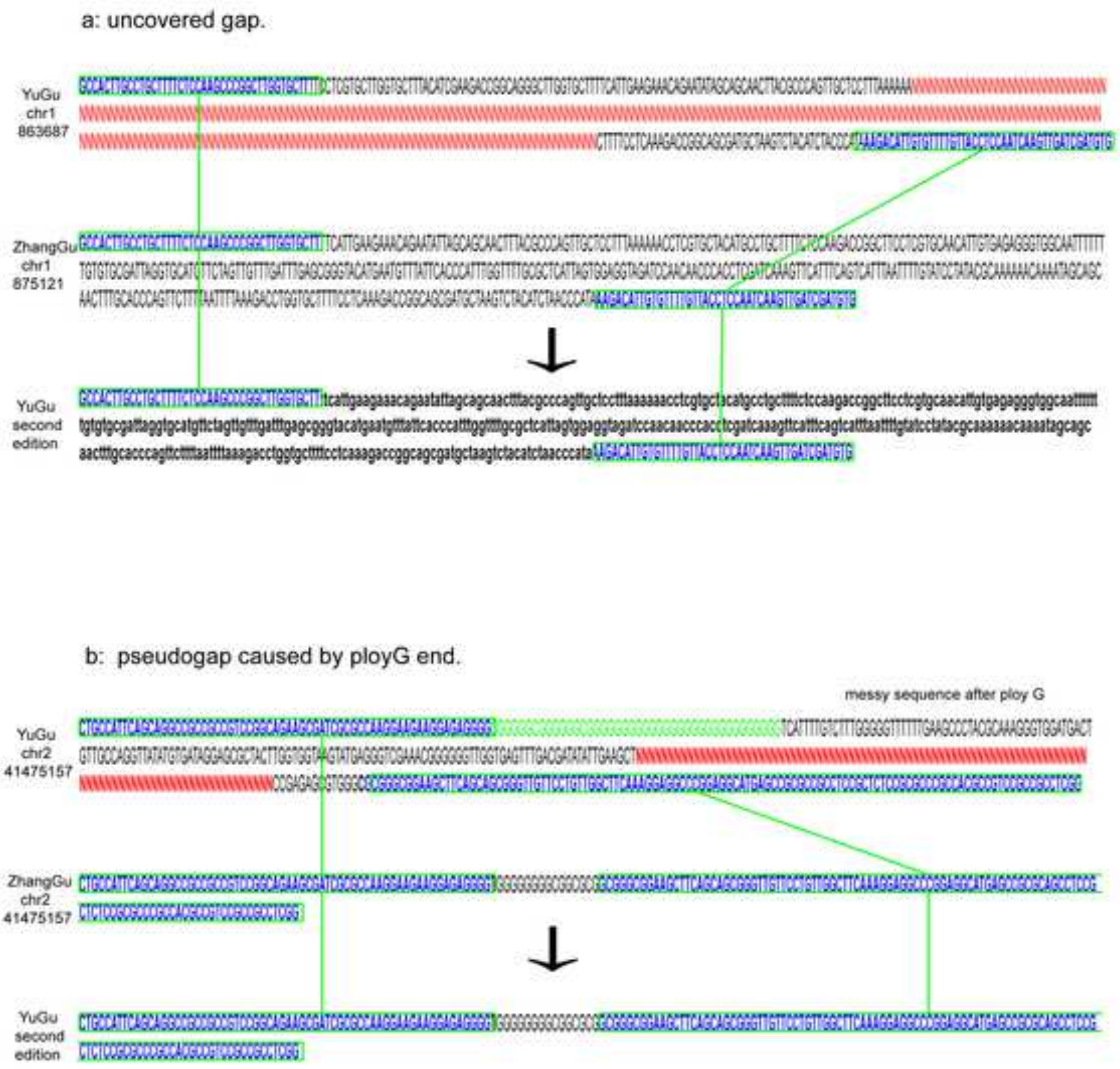

Figure4

Click here to download Figure Fig 4.tif

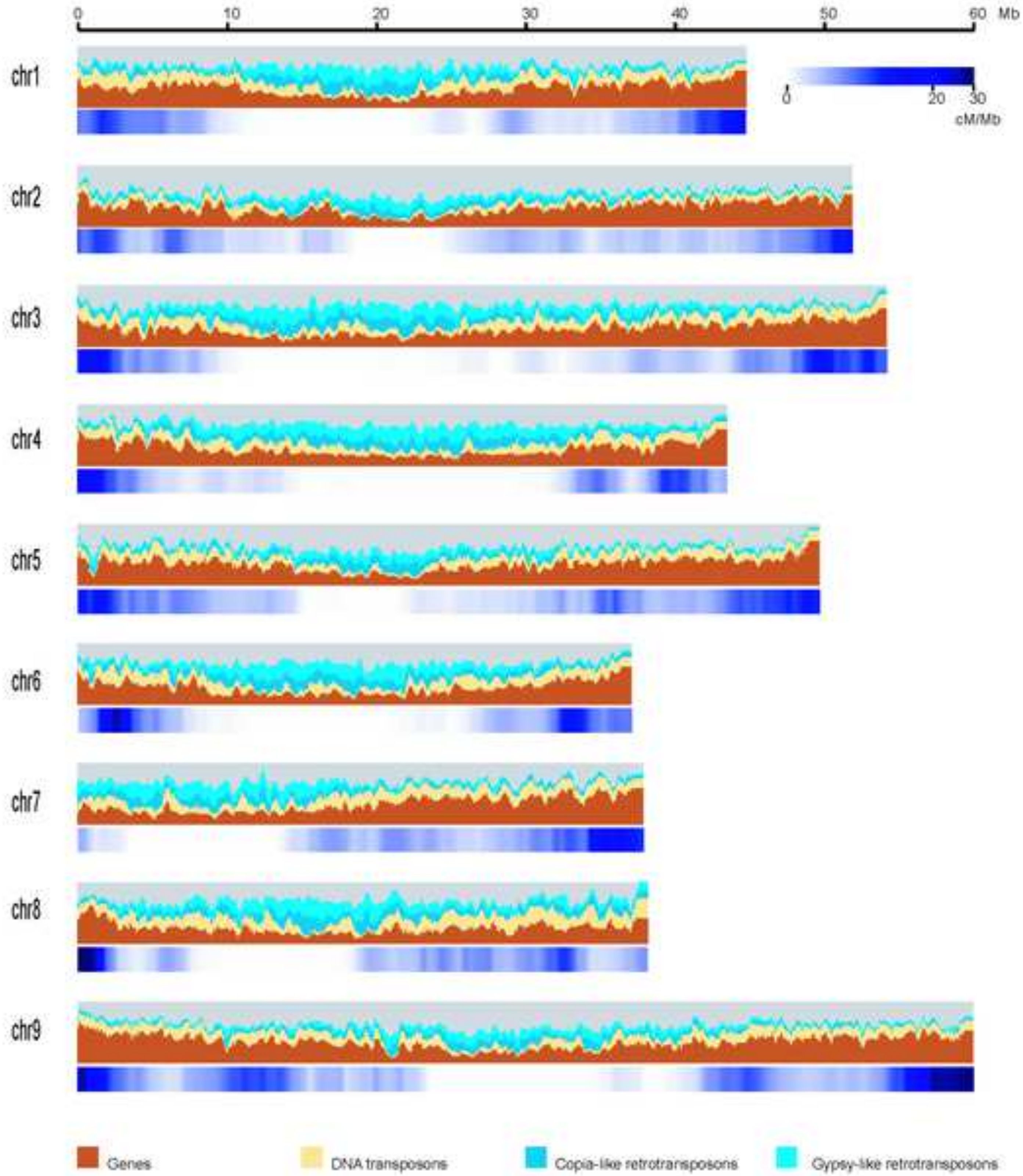

Figure5

Click here to download Figure Fig 5.tif

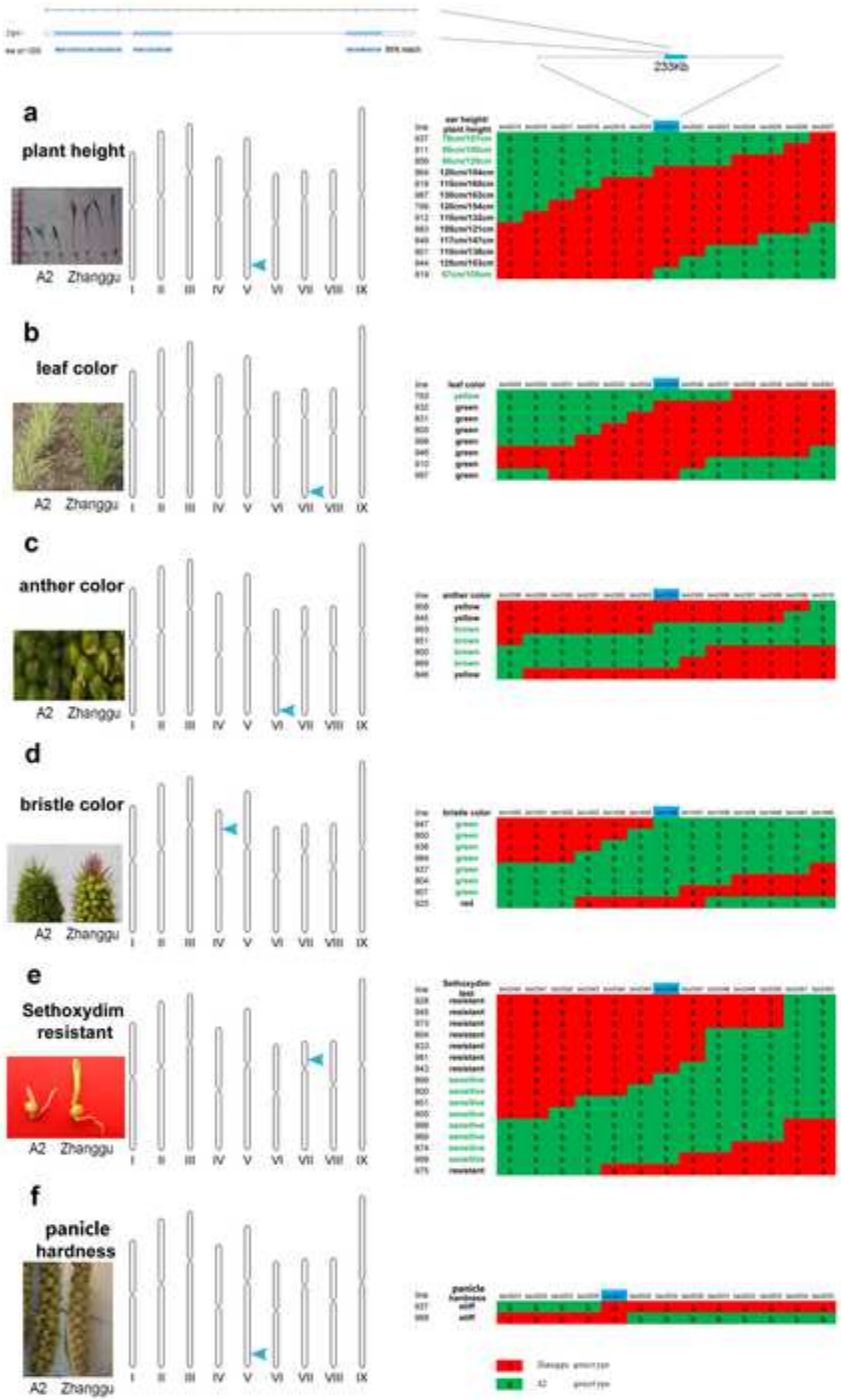

Figure6

Click here to download Figure Fig 6.jpg

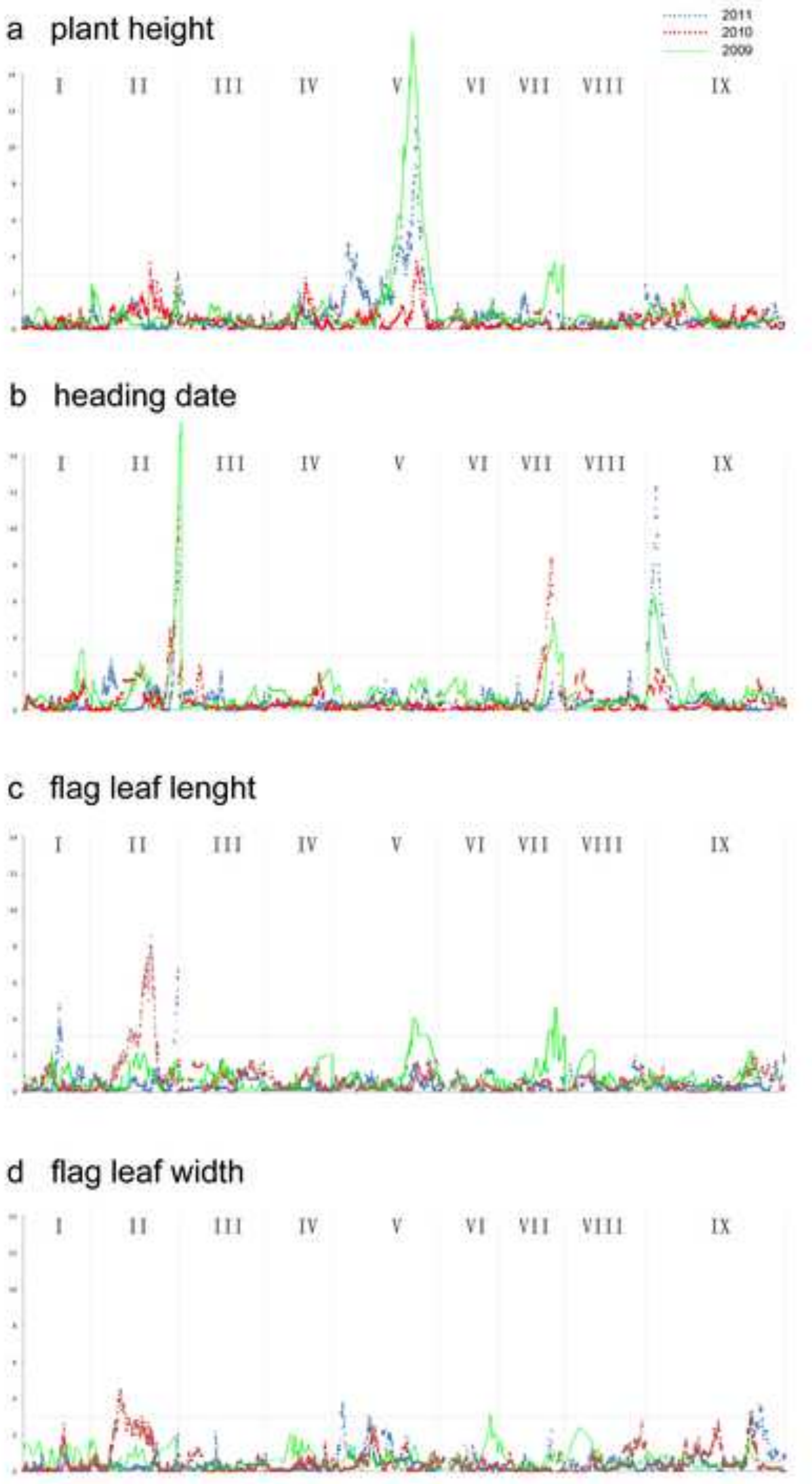

Supplement: GIGA-D-16-00059_Revision_1.pdf [file giw005_giga-d-16-00059_revision_1.pdf]
