# Supplementary material for: Updated foxtail millet genome assembly and gene mapping of nine key agronomic traits by resequencing a RIL population
Source: Gigascience. 2017 Jan 20;6(2):1–8. doi: 10.1093/gigascience/giw005 (PMC5466707; doi:10.1093/gigascience/giw005)
Supplement: Response_to_Reviewer_Comments_Original_Submission.pdf [file giw005_response_to_reviewer_comments_original_submission.pdf]

Reviewer #1:
The authors have developed a QTL mapping population for foxtail millet, created a dense genetic map using 184 RILs and mapped 9 agronomic traits. In general this is a solid work and suitable for publication. However, there are many writing issues with the article, in terms of language, organization and some details in methodology and results. Thus, I recommend the authors to go through the ms carefully for all the necessary corrections before its acceptance for publication. Some detail points are below:

1. p2l41-46. check grammar
2. p2l50. "de novel" should be all italic
3. p2l50. change finished to "initially finished". improvements are still needed for both genomes
4. p3l5. change "phenotype appraisal" to phenotyped
5. p3l7. delete "Gene mapping and", redudant with "QTL analysis"

Response: For Q1-5, we revised them.

6. p4l15. specify which generation of RIL did DNA extraction was done

Response: Leaf tissues of F10 was extracted, we added that.

7. p4l42. what is the average sequencing coverage?

Response: 140 Gb clean data was generated, which given a 2X depth coverage of each RIL. Please find it at the first line of the Result part.

8. p4l46. explain how this 11:4 was chosen

Response: This approach 11:4 was adopted in several researches and we followed their methods for genotyping identification (Huang et al. 2009; Duan et al. 2013; Wang et al. 2015).

9. p5l27. it is strange here - aren't the reads themselves originally mapped to Zhanggu genome? If so the reference alleles should be the Zhanggu allele?

Response: Yes, it is. Reads of all samples were mapped onto Zhanggu initial genome reference (containing 9 pseudo chromosomes and unmapped scaffolds) by using SOAP2 (LI et al. 2009) (vision 2.20).

10. p5l52-p6l21. this paragraph is redundant. It should be integrated into methods section.
p6l34. "184 RILs were aligned to a matrixs", rewrite. the sentence is not clear, also matrixs is a typo.

Response: We delete and revised it just as reviewer’s suggestion.

11. p7l15. keep font size consistent. How many replications were used?

Response: 1000 replications were used.

12. p7l19. change gene into loci. It is not confirmed yet the causal factor for the phenotype is a "gene"

Response: We do change “gene” into‟ loci”.

13. p7l20. define dominant here. explain why all loci are dominant?

Response: According to the phenotypes of parents and F1, these five traits were controlled by dominant genes.

14. p8l15. "Although foxtail millet was one of the most drought-tolerant crops, it was still substituted by

high yield bybrid corn." where? when? citation?

ResponsE: We change this sentence to “Although foxtail millet is one of the most drought-tolerant crops, its planting area declined sharply in the last 30 years in China, mainly substituted by high yield hybrid corn (http://www.nongtewang.org/grain/news/2016-01-07/50493.html).”

15. Figures. Figure resolution are pretty low. But I guess there should be a high res version for actual publication?

Response: We have uploaded more clear figures.

Reviewer #2:
1. Overall, this manuscript (MS) well written and has use full content/data for foxtail breeders but need for appropriate strategy to be discussed for iit utilization in the cultivar development program. Therefore, I recommend the following suggestion for MS improvement before accepting for better reading and breeder's usage.

2. Title should be changed to reflect the implication of the finding and database. 'Gene mapping of foxtail millet RIL populations for key agronomic traits"

Response: We change the title as suggested.

3. Abstract can improved with more finding and implications.

Response: Yes, we do rewrite 50% parts of the abstract part.

4. For uniformity in the manuscript use foxtail millet instead of millet (in some places)

Response: We revised them.

5. In materials and methods: Give some brief on trait recorded and then describe about how the traits were measured. Also Not clearly descried in phenotyping part.

Response: We revised large part of the materials and methods part, especially the Phenotyping , DNA isolation and genome sequencing, Sequence alignment, genotyping and recombination breakpoint determination and Yugu genome construction and gap filling parts.

6. Always simple language to understand the methods and nay description of terminology to readers.

Response: We revise most of the methods part in a more understandable way.

7. Tassel refers to panicle? Then use panicle as common in entire MS

Response: We have changed all “tassel” into “panicle”.

8. In result section: first para and second para few lines can be brought to m & M section as more reliable.

Response: Yes, we do change that.

9. In the result section, it is mentioned, New chromosomes were constructed based on bin map and scaffolds of Zhanggu, do authors refers to new linkage/genetic map? need clarification
Reads of all samples were mapped onto Zhanggu initial genome reference.

10. Fig. 6 b indicated that the leaf color also affect the heading date is it any report available to support this

statement?

Response: No, not as far as I know.

11. Authors missed to interpret this work closely towards breeding new cultivar and future of this map in foxtail improvement

Response: We have revised the discussion part and talking about the breeding future that could be used.

Reviewer #3: This is good work that improves the previously published assemblies using F2 population bin mapping. However, the following will need to be done:

1. The grammar and typo errors will need to be updated in the paper for the readers and reviewers to understand the paper properly

Response: We revise largely part of the MS with better language and grammar.

2. There are inconsistencies even in how parental genotypes are referred. For example, there is Yugu, Yuggu and YuGu? Are they the same thing?

Response: Yes, they are the same thing. We revised all of them as “Yugu”.

3. Do the authors mean drought-tolerant or drought resistant?

Response: We mean drought-tolerant, and change all the “drought resistant” into “drought-tolerant”.

4. There is really no real discussion of the paper. The section "discussion" is basically an extension of the "Results" section. For example, several candidate genes are listed including Z3lc, Z3sr, Z3bc, Z3ac, Z3th etc. But there is no discussion on what these genes are likely to be or whether similar genes have been identified in other crops.

Response: We have changed almost 80% part of discussion part, and added some Z3ph1 gene discussion contends.
