# Supplementary material for: Updated foxtail millet genome assembly and gene mapping of nine key agronomic traits by resequencing a RIL population
Source: Gigascience. 2017 Jan 20;6(2):1–8. doi: 10.1093/gigascience/giw005 (PMC5466707; doi:10.1093/gigascience/giw005)
Supplement: Reviewer_1_Reort_(Original_Submission).pdf [file giw005_reviewer_1_reort_(original_submission).pdf]

Reviewer Report

Title: "Updated foxtail millet genome assembly and gene mapping of nine key agronomic traits by resequencing a RIL population"

Version: Original Submission **Date:** 9/27/2016

Reviewer name: Pu Huang

Reviewer Comments to Author:

The authors have developed a QTL mapping population for foxtail millet, created a dense genetic map using 184 RILs and mapped 9 agronomic traits. In general this is a solid work and suitable for publication. However, there are many writing issues with the article, in terms of language, organization and some details in methodology and results. Thus, I recommend the authors to go through the ms carefully for all the necessary corrections before its acceptance for publication. Some detail points are below:p2l41-46. check grammarp2l50. "de novel" should be all italicp2l50. change finished to "initially finished". improvements are still needed for both genomesp3l5. change "phenotype appraisal" to phenotypedp3l7. delete "Gene mapping and", redudant with "QTL analysis"p4l15. specify which generation of RIL did DNA extraction was donep4l42. what is the average sequencing coverage?p4l46. explain how this 11:4 was chosenp5l27. it is strange here - aren't the reads themselves originally mapped to Zhanggu genome? If so the reference alleles should be the Zhanggu allele?p5l52-p6l21. this paragraph is redundant. It should be integrated into methods section.p6l34. "184 RILs were aligned to a matrixs", rewrite. the setence is not clear, also matrixs is a typo.p7l15. keep font size consistent. How many replications were used?p7l19. change gene into loci. It is not confirmed yet the causal factor for the phenotype is a "gene"p7l20. define dominant here. explain why all loci are dominant?p8l15. "Although foxtail millet was one of the most drought-tolerant crops, it was still substituted by high yield bybrid corn.". where? when? citation?Figures.Figure resolution are pretty low. But I guess there should be a high res version for actual publication?

Level of Interest

Please indicate how interesting you found the manuscript: An article of importance in its field

Quality of Written English

Please indicate the quality of language in the manuscript: Needs some language corrections before being published

Declaration of Competing Interests

Please complete a declaration of competing interests, considering the following questions:

- Have you in the past five years received reimbursements, fees, funding, or salary from an organisation that may in any way gain or lose financially from the publication of this manuscript, either now or in the future?
- Do you hold any stocks or shares in an organisation that may in any way gain or lose financially from the publication of this manuscript, either now or in the future?
- Do you hold or are you currently applying for any patents relating to the content of the manuscript?
- Have you received reimbursements, fees, funding, or salary from an organization that holds or has applied for patents relating to the content of the manuscript?
- Do you have any other financial competing interests?
- Do you have any non-financial competing interests in relation to this paper?

If you can answer no to all of the above, write 'I declare that I have no competing interests' below. If your reply is yes to any, please give details below.

I declare that I have no competing interests

I agree to the open peer review policy of the journal. I understand that my name will be included on my report to the authors and, if the manuscript is accepted for publication, my named report including any attachments I upload will be posted on the website along with the authors' responses. I agree for my report to be made available under an Open Access Creative Commons CC-BY license (http://creativecommons.org/licenses/by/4.0/). I understand that any comments which I do not wish to be included in my named report can be included as confidential comments to the editors, which will not be published.

I agree to the open peer review policy of the journal
