# Supplementary material for: Updated foxtail millet genome assembly and gene mapping of nine key agronomic traits by resequencing a RIL population
Source: Gigascience. 2017 Jan 20;6(2):1–8. doi: 10.1093/gigascience/giw005 (PMC5466707; doi:10.1093/gigascience/giw005)
Supplement: Reviewer_2_Report_(Original_Submission).pdf [file giw005_reviewer_2_report_(original_submission).pdf]

Reviewer Report

Title: "Updated foxtail millet genome assembly and gene mapping of nine key agronomic traits by resequencing a RIL population"

Version: Original Submission **Date:** 9/29/2016

Reviewer name: M Govindaraj

Reviewer Comments to Author:

1. Overall, this manuscript (MS) well written and has use full content/data for foxtail breeders but need for appropriate strategy to be discussed for iit utilization in the cultivar development program. Therefore, I recommend the following suggestion for MS improvement before accepting for better reading and breeder's usage. 2. Title should be changed to reflect the implication of the finding and database. 'Gene mapping of foxtail millet RIL populations for key agronomic traits",3. Abstract can improved with more finding and implications. 4. For uniformity in the manuscript use foxtail millet instead of millet (in some places) 5. In materials and methods: Give some brief on trait recorded and then describe about how the traits were measured. Also Not clearly descried in phenotyping part. 6. Always simple language to understand the methods and nay description of terminology to readers.7. Tassel refers to panicle? Then use panicle as common in entire MS8. In result section: first para and second para few lines can be brought to m & M section as more reliable.9. In the result section, it is mentioned, New chromosomes were constructed based on bin map and scaffolds of Zhanggu, do authors refers to new linkage/genetic map ? need clarification 10. Fig. 6 b indicated that the leaf color also affect the heading date is it any report available to support this statement? 11. Authors missed to interpret this work closely towards breeding new cultivar and future of this map in foxtail improvement

Level of Interest

Please indicate how interesting you found the manuscript: An article of importance in its field

Quality of Written English

Please indicate the quality of language in the manuscript: Needs some language corrections before being published

Declaration of Competing Interests

Please complete a declaration of competing interests, considering the following questions:

- Have you in the past five years received reimbursements, fees, funding, or salary from an organisation that may in any way gain or lose financially from the publication of this manuscript, either now or in the future?

- Do you hold any stocks or shares in an organisation that may in any way gain or lose financially from the publication of this manuscript, either now or in the future?
- Do you hold or are you currently applying for any patents relating to the content of the manuscript?
- Have you received reimbursements, fees, funding, or salary from an organization that holds or has applied for patents relating to the content of the manuscript?
- Do you have any other financial competing interests?
- Do you have any non-financial competing interests in relation to this paper?

If you can answer no to all of the above, write 'I declare that I have no competing interests' below. If your reply is yes to any, please give details below.

I declare that I have no competing interests

I agree to the open peer review policy of the journal. I understand that my name will be included on my report to the authors and, if the manuscript is accepted for publication, my named report including any attachments I upload will be posted on the website along with the authors' responses. I agree for my report to be made available under an Open Access Creative Commons CC-BY license (http://creativecommons.org/licenses/by/4.0/). I understand that any comments which I do not wish to be included in my named report can be included as confidential comments to the editors, which will not be published.

I agree to the open peer review policy of the journal
