# Supplementary material for: Updated foxtail millet genome assembly and gene mapping of nine key agronomic traits by resequencing a RIL population
Source: Gigascience. 2017 Jan 20;6(2):1–8. doi: 10.1093/gigascience/giw005 (PMC5466707; doi:10.1093/gigascience/giw005)
Supplement: Reviewer_3_Report_(Original_Submission).pdf [file giw005_reviewer_3_report_(original_submission).pdf]

Reviewer Report

Title: "Updated foxtail millet genome assembly and gene mapping of nine key agronomic traits by resequencing a RIL population"

Version: Original Submission **Date:** 10/4/2016

Reviewer name: Damaris Odeny

Reviewer Comments to Author:

Click here to enter text.

This is good work that improves the previously published assemblies using F2 population bin mapping. However, the following will need to be done:

1. The grammar and typo errors will need to be updated in the paper for the readers and reviewers to understand the paper properly

2. There are inconsistencies even in how parental genotypes are referred. For example, there is Yugu, Yuggu and YuGu? Are they the same thing?

3. Do the authors mean drought-tolerant or drought resistant?

4. There is really no real discussion of the paper. The section "discussion" is basically an extension of the "Results" section. For example, several candidate genes are listed including Z3lc, Z3sr, Z3bc, Z3ac, Z3th etc. But there is no discussion on what these genes are likely to be or whether similar genes have been identified in other crops.

Level of Interest

Please indicate how interesting you found the manuscript: An article of importance in its field

Quality of Written English

Please indicate the quality of language in the manuscript: Not suitable for publication unless extensively edited

Declaration of Competing Interests

Please complete a declaration of competing interests, considering the following questions:

- Have you in the past five years received reimbursements, fees, funding, or salary from an organisation that may in any way gain or lose financially from the publication of this manuscript, either now or in the future?
- Do you hold any stocks or shares in an organisation that may in any way gain or lose financially from the publication of this manuscript, either now or in the future?

- Do you hold or are you currently applying for any patents relating to the content of the manuscript?
- Have you received reimbursements, fees, funding, or salary from an organization that holds or has applied for patents relating to the content of the manuscript?
- Do you have any other financial competing interests?
- Do you have any non-financial competing interests in relation to this paper?

If you can answer no to all of the above, write 'I declare that I have no competing interests' below. If your reply is yes to any, please give details below.

I declare that I have no competing interests

I agree to the open peer review policy of the journal. I understand that my name will be included on my report to the authors and, if the manuscript is accepted for publication, my named report including any attachments I upload will be posted on the website along with the authors' responses. I agree for my report to be made available under an Open Access Creative Commons CC-BY license (http://creativecommons.org/licenses/by/4.0/). I understand that any comments which I do not wish to be included in my named report can be included as confidential comments to the editors, which will not be published.

I agree to the open peer review policy of the journal
